# Corneal ulcers in children

**Published:** 2024-02-09

**Authors:** Clare Gilbert

**Affiliations:** 1 Professor of International Eye Health: International Centre for Eye Health, London School of Hygiene & Tropical Medicine, London, UK.

**It is vital to recognise and urgently treat corneal ulcers in children, as they can lead to permanent vision impairment. Children with corneal ulcers due to vitamin A deficiency also have an extremely high risk of mortality.**

**Figure:**
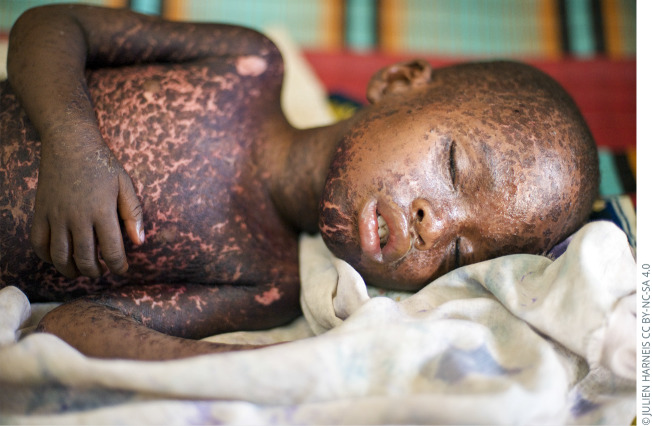
Measles infection is associated with corneal ulcers due to vitamin A deficiency, herpes simplex virus, and the use of traditional remedies. It is becoming more common due to declining immunisation coverage worldwide. guinea

Athough corneal ulcers can affect people of all ages, it is particularly important to recognise and treat them urgently in children, as ulcers can lead to scarring and complications that may impair a child's vision permanently. Children with corneal ulcers due to severe vitamin A deficiency are not only at risk of impaired vision, but also have an extremely high mortality rate: 60% are likely to die within 3 months, unless they are treated.

Other causes of corneal ulcers include injury/trauma, use of harmful remedies, autoimmune conditions, and infections due to viruses, bacteria, fungi, and protozoa. In this article, we focus on causes that are specific to children. For more information on managing corneal ulcers that affect people at any age, including those caused by bacteria and fungi, visit the *Community Eye Health Journal* online (www.cehjournal.org) and use the search function.

## What to look for and do at community/primary level

### Red eye and suspected corneal ulcer

Red eyes are common in children, and most resolve with appropriate care. However, the presence of one or more of these signs suggest that a child has developed a corneal ulcer and needs urgent treatment:

- The eye is watering excessively
- The child shows signs of being in pain
- The child shows signs of being sensitive to light
- The child can see less well (measurable as a decrease in visual acuity).

**What to do:** Immediately refer these children to an eye department where there is an ophthalmologist. If the child is up to 1 month of age, and has swelling of the eyelids as well as profuse, purulent discharge, this could be conjunctivitis of the newborn due to gonococcal infection (see page 4). Administer intramuscular antibiotics, if available (ceftriaxone 25–50 mg/kg, up to a maximum of 125 mg), and refer the child urgently.

### Vitamin A deficiency

Corneal ulcers due to vitamin A deficiency do not always cause a red eye, and are not painful (they may cause discomfort, however, and the child may keep their eyes closed). Suspect corneal ulcers due to vitamin A deficiency if:

- There is a recent or current history of fever, measles, or diarrhoea
- Both eyes are affected.

**What to do:** Immediately administer high-dose vitamin A (retinyl palmitate) and refer the child to a hospital. Follow up in 24 hours to ensure they are getting the help they need.

The recommended dose for each age group is:

- < 6 months of age: 50,000 international units (IU)
- 6–12 months: 100,000 IU
- > 1 year: 150,000 IU

Children must receive three doses of vitamin A: on day 1, day 2, and day 14.

## Diagnosis and management in the eye department

The table below lists the most common causes of corneal ulcers in children, alongside the age typically affected, the organism responsible (if any), and the history, diagnosis, and management of each condition.

## Prevention of corneal ulcers in children

Many of the causes of corneal ulcers in children can be prevented as follows:

1. Ocular prophylaxis of the newborn (cleaning the eyelids immediately after birth and instilling a topical antibiotic or antiseptic
2. Measles immunisation and vitamin A supplementation.
3. Good hand hygiene and avoiding getting water in the eyes of contact lens wearers.
4. Preventing the use of harmful eye remedies, which requires measures such as health education and improved access to eye care services.

**Table:** Common causes of corneal ulcers in children: history, diagnosis and management

**Cause of the ulcer**	**Age of child**	**Infection?**	**History**	**Diagnosis**	**Management**
**Conjunctivitis of the newborn (ophthalmia neonatorum)**( **Figure:** 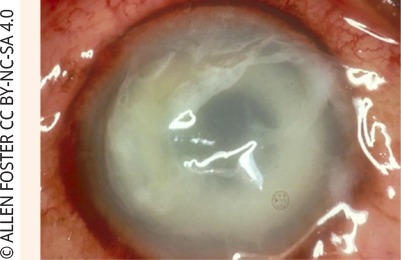 Corneal ulcer and abscess in gonococcal ophthalmia neonatorum. tanzania	< 1 month	Ophthalmia neonatorum can be due to a range of organisms, but only gonococcal infection causes corneal ulcers and corneal abscesses.	Lid swelling and profuse, purulent discharge	Conjunctival swab with microscopy (Gram stain)	If gonococcal infection is confirmed, give ceftriaxone as a single dose of 25–50 mg/kg intramuscular or intravenous, up to a maximum of 125 mg. The mother and her sexual partner also need to be treated.
**Corneal infection (microbial keratitis)** **Figure:** 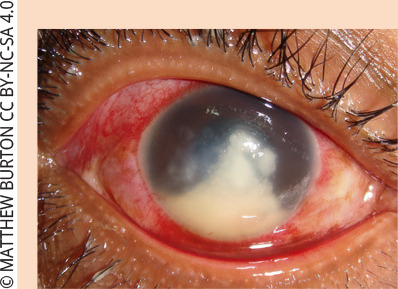 Often associated with trauma to the eye and usually caused by fungi or bacteria, with *Acanthamoeba* sp. a less common cause.	Any age	Yes – bacteria, fungi and/or protozoa (*Acanthamoeba* sp.)	Trauma to the eye, e.g. with plant matter, dust, use of topical traditional medicines or poor contact lens hygiene.	Corneal scrape with microscopy (Gram stain) and culture. Suspect *Acanthamoeba* sp. if culture is negative for bacteria and fungi and there is a history and clinical signs suggestive of this type of infection; positive diagnosis is challenging, requiring PCR or confocal microscopy.	Intensive topical treatment, guided by laboratory results Read more here: Clinical diagnosis and management http://tinyurl.com/mkman Taking a corneal scrape http://tinyurl.com/mkscrape
**Vitamin A deficiency/measles** **Figure:** 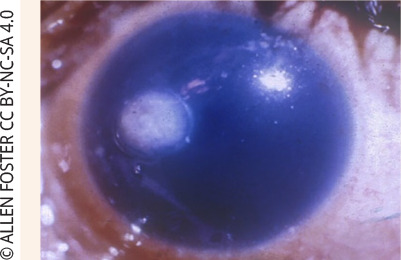 Corneal ulcer due to acute vitamin A deficiency. Note that the cornea is dry and slightly hazy, and the eye is not very inflamed. tanzania	< 5 years	No. There may be corneal melting (keratomalacia)	Fever, measles, diarrhoea, inadequate nutrition, (often associated with poverty)	Clinical suspicion/medical history	High-dose vitamin A Topical antibiotics if there is secondary infection
**Harmful eye remedies** **Figure:** 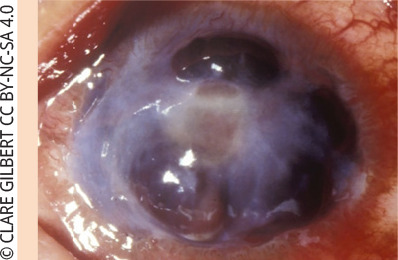 Atypical corneal ulcer in a girl treated using her uncle's urine, which was infected with *Neisseria gonorrhoeae*. sierra leone	Any age	Depends on what was put in the eye(s)	Measles infection or conjunctivitis. Note: carers may not give a history of use	Corneal scrape with microscopy (Gram stain) and culture	Intensive topical treatment, guided by laboratory results
**Herpes simplex** **Figure:** 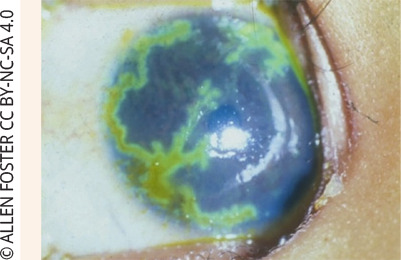 Dendritic ulcer due to Herpes simplex stained with fluorescein. tanzania	Any age	Yes, viral infection	Recent measles infection. May be recurrent.	Clinical diagnosis	Treat using topical antiviral agents, such as acyclovir and ganciglovir. Close follow-up is needed.
**Severe vernal keratoconjunctivitis** **Figure:** 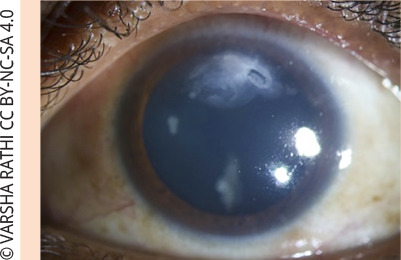 Shield ulcer complicating severe vernal keratoconjunctivitis. india	Older children/adolescents	No	Chronic irritation, watering with stringy discharge May have other allergies such as eczema or asthma	Clinical examination for typical signs of vernal keratoconjuntivitis: Limbal papillae Cobblestone or giant papillae respectively Scarring of the upper tarsal plate. See previous CEHJ article about vernal keratoconjuctivitis: http://tinyurl.com/yc6wm8px	Shield ulcers can be graded depending on their severity:^1^ **Grade 1** An ulcer with a clear/transparent base. Usually responds to medical treatment alone (to control inflammation and allow the cornea to heal). Topical anti-inflammatory eye drops including sodium chromoglycate and topical cyclosporin can be used. Topical steroids are not advised due to the risk of secondary cataract and glaucoma; however, subtarsal injection of long-acting, depot steroids can be used. **Grade 2** An ulcer with a translucent base (i.e., it lets some light through), with or without opaque white or yellow deposits (inflammatory debris). The deposits filling the ulcer need to be gently removed and the eye padded. **Grade 3** An ulcer which has elevated plaque formation. These ulcers may need an amniotic membrane graft to promote healing.
